# Supplementary material for: Transcriptomics Integrated with Metabolomics: Assessing the Central Metabolism of Different Cells after Cell Differentiation in Aureobasidium pullulans NG
Source: J Fungi (Basel). 2022 Aug 22;8(8):882. doi: 10.3390/jof8080882 (PMC9410427; doi:10.3390/jof8080882)
Supplement: Supplementary file 1 [file jof-08-00882-s001.zip › jof-1827445-supplementary.pdf]

**Table S1** primers for this study.

| Primer name | Primer sequence(5′-3′) |
|---|---|
| SC16908_c1_g2-F | GTCCGTGATATTCGTGGCTGGTC |
| SC16908_c1_g2-R | TGTGAGAACGGTTGACGCTTGG |
| SC16627_c2_g5-F | TGCTCCTCGTCGTACACCTATCTAC |
| SC16627_c2_g5-R | TACTGCGTTGACCTTCCAAGTTGTG |
| SC2555_c0_g1-F | GCTGTATGCGGACAAGGATCTGG |
| SC2555_c0_g1-R | GGTGAGTCAACGGTTAGCCATATCG |
| SC16598_c2_g1-F | TCGCCTGAACGCATTGAGAACG |
| SC16598_c2_g1-R | GACTCCGATGACAGCAACGAGATAC |
| SC16876_c1_g2-F | TCATTGGAAAGGTCGTCAAGGTTGG |
| SC16876_c1_g2-R | AGTAGTTCTCTTGGTCGGCATTGC |
| SC16337_c0_g3-F | CGACAAGGTCATCAACGACAAGTTC |
| SC16337_c0_g3-R | TCTTATGGGTGGCGGTGTAGGAG |
| SC16350_c0_g1-F | TGACCACCATCCACTCCTACACC |
| SC16350_c0_g1-R | AATGACCTTGCCGACAGCCTTG |
| SC14418_c0_g3-F | CGAATGCGGGTCCTCTGTCATG |
| SC14418_c0_g3-R | GACGAAACACGCTGGTCTGGAG |
| SC16410_c0_g3-F | CGCTCTCTCCTCTCCTTTCCTCTC |
| SC16410_c0_g3-R | GCCAACCTCAGCCTTCATCTCAG |
| SC16898_c5_g17-F | CTTGAGCGTGACGAGACTCTTAGC |
| SC16898_c5_g17-R | TTGCGGTTGTAGGAAGTGAAGATGG |

Table S2 Candidate genes involved in antioxidation in SC.

| Description | Gene ID | KEGG pathway | Expressd level |
|---|---|---|---|
| CAT1 catalase | SC16638_0_1 | Glyoxylate and dicarboxylate | UP |
| | SC16638_0_2 | metabolism | UP |
| | SC16638_0_3 | | UP |
| S-2-hydroxy-acid | SC4646_0_1 | Glyoxylate and dicarboxylate | UP |
| oxidase-like protein | | metabolism | |
| catalase-domain-containing | SC16908_1_2 | Glyoxylate and dicarboxylate | UP |
| protein | SC16908_1_1 | metabolism | UP |
| isocitrate lyase and | SC16145_0_1 | Glyoxylate and dicarboxylate | UP |
| phosphorylmutase | SC16312_3_7 | metabolism | UP |
| CAT2 catalase | SC16627_2_5 | Glyoxylate and dicarboxylate | UP |
| | SC16627_2_3 | metabolism | UP |
| malate dehydrogenase | SC16905_3_2 | Pyruvate metabolism | UP |
| | SC16905_3_1 | | UP |
| | SC15936_0_2 | Glycolysis / Gluconeogenesis | UP |
| malate synthase | SC13547_0_1 | Glyoxylate and dicarboxylate | UP |
| | | metabolism | |
| ATP-utilizing | SC16713_6_1 | Glycolysis / Gluconeogenesis | UP |
| phosphoenolpyruvate | SC16713_4_4 | | UP |
| carboxykinase | SC16713_4_1 | | UP |

| | | | |
|---|---|---|---|
| | SC16713_4_6 | Pyruvate metabolism | UP |
| L-lactate dehydrogenase | SC2555_0_1 | Pyruvate metabolism | UP |
| acyl-CoA oxidase | SC16549_2_1 | Fatty acid degradation | UP |
| | SC16598_2_1 | | UP |
| | SC16641_0_4 | | UP |
| | SC15352_1_1 | | UP |
| alcohol dehydrogenase | SC16055_0_2 | Glycolysis / Gluconeogenesis | UP |
| | SC16055_0_1 | | UP |
| | SC16341_0_6 | | UP |
| | SC16876_1_2 | | UP |
| fructose-BISphosphate | SC15595_0_2 | Pentose phosphate pathway | UP |
| ALDOLASE， class II | SC15595_0_1 | Pentose phosphate pathway | UP |
| | SC15595_0_2 | Fructose and mannose metabolism | UP |
| fructose-1，6-bisphosphatase | SC16924_2_1 | Fructose and mannose metabolism | UP |
| glyceraldehyde-3-phosphate | SC16337_0_3 | Glycolysis / Gluconeogenesis | UP |
| dehydrogenase | SC16350_0_1 | | UP |
| alpha-amylase | SC14418_0_3 | Starch and sucrose metabolism | UP |
| | SC14418_0_1 | | |

Table S3 Differential metabolite KEGG enrichment results in SC.

| KEGG pathway | Total | Hits | Raw p | -LOG(p) |
|---|---|---|---|---|
| Nitrogen metabolism | 8 | 3 | 0.20697 | 1.5752 |
| beta-Alanine metabolism | 7 | 2 | 0.42941 | 0.84535 |
| Valine, leucine and isoleucine degradation | 16 | 4 | 0.41035 | 0.89074 |
| Arginine and proline metabolism | 37 | 8 | 0.48088 | 0.73214 |
| Tyrosine metabolism | 19 | 4 | 0.55568 | 0.58755 |
| Lysine biosynthesis | 19 | 4 | 0.55568 | 0.58755 |
| Propanoate metabolism | 14 | 3 | 0.56118 | 0.57771 |
| Nicotinate and nicotinamide metabolism | 9 | 2 | 0.5712 | 0.56001 |
| Pyrimidine metabolism | 35 | 7 | 0.58235 | 0.54068 |
| Alanine, aspartate and glutamate | 20 | 4 | 0.59983 | 0.51112 |

| | | | | |
|---|---|---|---|---|
| metabolism | | | | |
| Pantothenate and CoA biosynthesis | 16 | 3 | 0.65809 | 0.41841 |
| Tryptophan metabolism | 27 | 5 | 0.6654 | 0.40736 |
| Phenylalanine, tyrosine and tryptophan biosynthesis | 22 | 4 | 0.67981 | 0.38593 |
| Vitamin B6 metabolism | 11 | 2 | 0.68579 | 0.37718 |
| Purine metabolism | 60 | 11 | 0.69879 | 0.3584 |
| Pentose and glucuronate interconversions | 12 | 2 | 0.73304 | 0.31055 |
| Valine, leucine and isoleucine biosynthesis | 24 | 4 | 0.74804 | 0.2903 |
| Citrate cycle (TCA cycle) | 20 | 3 | 0.80358 | 0.21868 |
| Glycine, serine and threonine metabolism | 26 | 4 | 0.80466 | 0.21733 |
| Glyoxylate and dicarboxylate metabolism | 14 | 2 | 0.80964 | 0.21117 |
| Lysine degradation | 16 | 2 | 0.8661 | 0.14376 |
| Glutathione metabolism | 23 | 3 | 0.8753 | 0.13318 |
| Cyanoamino acid metabolism | 10 | 1 | 0.89681 | 0.10891 |
| Pentose phosphate pathway | 18 | 2 | 0.90687 | 0.097757 |
| Thiamine metabolism | 11 | 1 | 0.91791 | 0.085654 |
| Riboflavin metabolism | 11 | 1 | 0.91791 | 0.085654 |
| Inositol phosphate metabolism | 19 | 2 | 0.92262 | 0.080542 |
| Cysteine and methionine metabolism | 33 | 4 | 0.9273 | 0.075482 |
| Sulfur metabolism | 13 | 1 | 0.9481 | 0.053296 |
| Aminoacyl-tRNA | 67 | 9 | 0.95052 | 0.050746 |

| | | | | |
|---|---|---|---|---|
| biosynthesis | | | | |
| Amino sugar and nucleotide sugar metabolism | 24 | 2 | 0.97026 | 0.030188 |
| Fructose and mannose metabolism | 17 | 1 | 0.97933 | 0.020888 |
| Butanoate metabolism | 17 | 1 | 0.97933 | 0.020888 |
| Steroid biosynthesis | 23 | 1 | 0.99485 | 0.005161 |
| Glycolysis or Gluconeogenesis | 24 | 1 | 0.99592 | 0.0040869 |
| Glycerophospholipid metabolism | 26 | 1 | 0.99744 | 0.0025613 |
| Fatty acid metabolism | 28 | 1 | 0.9984 | 0.0016035 |
| Biosynthesis of unsaturated fatty acids | 42 | 2 | 0.99928 | 0.00072046 |
| Fatty acid biosynthesis | 37 | 1 | 0.99981 | 0.00019215 |

Note:Total, total number of metabolites in the target metabolic pathway; Hits, number of differential metabolites in the target metabolic pathway, Raw p, p-value of the hypergeometric distribution test; -log(p): negative value for the natural logarithm of the p-value.
